# Toward a Definition of the Linguistic Profile of Children With Autism Spectrum Disorder

**DOI:** 10.3389/fpsyg.2020.00808

**Published:** 2020-05-05

**Authors:** Andrea Marini, Martina Ozbič, Rita Magni, Giovanni Valeri

**Affiliations:** ^1^Department of Languages, Literatures, Communication, Education and Society, University of Udine, Udine, Italy; ^2^Scientific Institute IRCCS “Eugenio Medea”, San Vito al Tagliamento, Italy; ^3^Department of Neuroscience, Bambino Gesù Children’s Hospital, IRCCS, Rome, Italy

**Keywords:** Autism Spectrum Disorder, language, narrative analysis, developmental neuropsychology, neurolinguistics

## Abstract

The current investigation assessed linguistic and narrative abilities in a cohort of children with Autism Spectrum Disorder (ASD). The linguistic assessment was performed with both traditional tests and a multilevel procedure for discourse analysis. The results showed difficulties at different stages of message planning, organization, and microlinguistic processing (i.e., lexical selection and grammatical processing). Their macrolinguistic impairments were likely related to more general difficulties in the prelinguistic conceptual phase of message planning and mental model generation. Such weaknesses included a difficulty in the non-verbal conceptualization of the story and the generation of an internal representation of the addressee’s mental model.

## Introduction

Autism spectrum disorder (ASD) is characterized by persistent deficits in social communication and interaction associated with restricted and repetitive patterns of behavior, interests or activities (American Psychiatric Association, 2013). Because of its pivotal role in communicative interactions, since the seminal descriptions provided by Kanner (1943) and Asperger (1944/1991) language development and functioning in ASD has been the focus of extensive research (see also Boucher, 2012 for a comprehensive review). However, an accurate linguistic assessment of individuals with ASD must consider the actual complexity of the linguistic system. Language can be assessed from a micro- and a macrolinguistic perspective (Glosser and Deser, 1990; Marini et al., 2011): the microlinguistic perspective focuses on the intra-sentential (i.e., within-utterance) organization of discourse by assessing the phonetic, phonological and morphological skills needed to process words (lexical processing) and the morphosyntactic and syntactic abilities involved in the generation of sentences (syntactic processing); the macrolinguistic perspective focuses on the inter-sentential (i.e., between-utterances) processing by assessing the ability to select contextually appropriate words and utterances (pragmatic processing) and to generate cohesive and coherent ties among the sentences (discourse processing; Kintsch, 1994).

Longitudinal studies on language development in ASD have shown that the linguistic profiles of these children might change significantly with age (Bennett et al., 2008; Geurts and Embrechts, 2008; Rapin et al., 2009). Preschoolers are most likely to show phonological (but not necessarily articulatory) and grammatical impairments. For example, Tuchman et al. (1991) reported that in a cohort of 197 children with ASD, 117 individuals (59%) showed phonological and grammatical difficulties. Similarly, Allen and Rapin (1992) showed that all the individuals in a cohort of 229 preschoolers with ASD aged between 4 and 5 years showed not only pragmatic impairments but also some difficulties in linguistic comprehension. Sixty-three percent of these children (*N* = 144) experienced also phonological and syntactic impairments. These two studies had partially overlapping cohorts of individuals. However, grammatical impairments have been observed also in different groups of preschoolers with ASD (e.g., Eigsti et al., 2007). If phonological and grammatical impairments are frequent in preschoolers, pragmatic disturbances predominate by school-age (Geurts and Embrechts, 2008).

While informative and interesting, studies on language in ASD have usually focused on single aspects of language processing without considering it in its complexity. At times, this has led to mixed results. Indeed, even if pragmatic and discourse difficulties are a common finding in individuals with ASD, not all of them show phonological, lexical and/or grammatical difficulties. Even those who experience these symptoms may exhibit large within-group variability (e.g., Rapin and Dunn, 2003). In the 80’s, such observations led to the exclusion of language impairments from the criteria for the diagnosis of ASD and prompted a gradual shift of attention from the description of the linguistic features of the general population of individuals with ASD taken as a whole to more focused analyses of the linguistic characteristics of specific subgroups with linguistic impairment or delay (e.g., Kjelgaard and Tager-Flusberg, 2001; Tager-Flusberg and Joseph, 2003; Whitehouse et al., 2008). For example, Kjelgaard and Tager-Flusberg (2001) administered a range of language tests to 89 children with ASD aged 4–14 years. They were highly heterogeneous. Indeed, according to their performance on a test of lexical comprehension, the authors managed to cluster them in three major subgroups: one with normal linguistic performance (Autistic individuals with Normal Language, ALN); one with borderline performance and scores ranging within 1 and 2 standard deviations below the mean (Autistic individuals with borderline language skills); one with overtly impaired performance (Autistic individuals with Language Impairment, ALI). Articulation was normal in the ALN and borderline subgroups and mildly impaired in the ALI population. However, all three subgroups experienced difficulties on tasks assessing lexical comprehension and production (especially the ALI population) with the most important difficulties on tasks assessing pragmatic skills. Importantly, in this study the performance on a task of non-word repetition proved highly sensitive to the presence of linguistic disturbances: only the ALI subgroup was found significantly impaired. Subsequent studies focusing on school-age children with ALI ranging from 6 years up highlighted persisting morphological difficulties (Roberts et al., 2004) often characterized by the omission or substitution of function words (e.g., prepositions, articles or conjunctions; Lai, 2011). In spontaneous language, these difficulties may lead to reduced mean length of utterance (MLU; measured in morphemes as in Condouris et al., 2003) and syntactic structures that are fewer (e.g., Lai, 2011) and less variable (e.g., Losh and Capps, 2003) than normal. This is interesting, as often no syntactic difficulties are noticeable when their grammatical skills are assessed in more structured and decontextualized tests (e.g., Shulman and Guberman, 2007).

Traditional tests cannot adequately describe the linguistic profile of children with communicative disorders (e.g., Marini et al., 2008; Volden et al., 2017). To capture the interactions between different linguistic skills, it is necessary to include also procedures of narrative discourse assessments (Marini et al., 2005, 2014). Indeed, the generation of an informative message requires the speaker to consider the context and tie the different propositions through cohesive and coherent links. Therefore, a comprehensive assessment cannot be limited to the analysis of the microlinguistic features of message production but must include also the macrolinguistic ones (Volden et al., 2017). Overall, the narrative language produced by children with ASD has been described as idiosyncratic at both micro- and macrolinguistic levels of processing (e.g., Baixauli et al., 2016). Microlinguistic difficulties include the production of utterances characterized by unusual words, aberrant prosodic contours, and instances of pronoun reversal (e.g., Kuijper et al., 2017) with anomalous productivity levels and grammatical structuring (Baixauli et al., 2016). Macrolinguistic difficulties include the production of speech samples that are perceived as contextually inappropriate for the inclusion of echolalic, repetitive and overtly incoherent utterances (e.g., Kuijper et al., 2017). Furthermore, they have significant difficulties in the production of appropriately informative referring expressions (e.g., Arnold et al., 2009; Banney et al., 2015). As to this regard, a recent investigation by Malkin et al. (2018) showed that, even if they can take to some extent the interlocutor-specific prior experience into account, children with ASD may lag behind typical peers in the degree to which they make use of such information. Difficulties have been reported in the ability to establish causal connections between the utterances (e.g., Diehl et al., 2006; Baixauli et al., 2016; Volden et al., 2017) and organize the temporal dynamics of narrative discourse (Ferretti et al., 2018; Marini et al., 2019) to the extent that they are often not able to adequately use story-grammar information to organize their narrative speech samples (Goldman, 2008; McCabe et al., 2013).

As it is evident from this brief analysis of the available literature, linguistic skills in ASD have been widely explored. However, some issues remain unresolved. First, it is not clear yet whether a morphological difficulty can be ascribed to persons with ASD and language impairment and whether it is related to their grammatical skills while producing a narrative discourse. For example, the already mentioned study by Roberts et al. (2004) suggests that difficulties in verb tense might be an important marker of the linguistic symptomatology observable in these children. Evidence of morphological difficulties leading to omissions of function words further supports this possibility (e.g., Botting and Conti-Ramsden, 2003; Condouris et al., 2003). However, to the best of our knowledge, no study has explicitly explored the possible relation between the morphological impairments often observed in ALI children and grammatical (i.e., morphosyntactic and syntactic) difficulties in discourse production. In our view, one further aspect requires explicit analysis: the possibility that different types of macrolinguistic difficulties are related to the microlinguistic impairments observable on a narrative production task. Consequently, this study aimed to replicate and expand upon previous research on both micro- and macrolinguistic skills in a group of Italian-speaking school-age children with ASD and microlinguistic impairment (ALI). Namely, to have a detailed profile of their linguistic and narrative skills we jointly adopted traditional standardized procedures for linguistic analysis and a multilevel procedure for discourse analysis that has proven useful in detecting micro- and macrolinguistic impairments in both children and adult patients with communicative disorders (e.g., Marini et al., 2010, 2014). We assumed that this accurate analysis would allow us to efficiently describe the micro- and macrolinguistic abilities of the children with ALI and provide additional information about these features in children with a language, Italian, that is structurally dissimilar from English. Furthermore, as it enables the exploration of the complex interactions between micro- and macrolinguistic processes, we hypothesized that the multilevel procedure for discourse analysis would highlight the potential effect of macrolinguistic variables on microlinguistic (i.e., lexical and sentence-level processing) performance. In particular, it was hypothesized that selective problems in microlinguistic processing would be related to a more general problem in discourse planning and organization.

## Methods

### Participants

Seventy-four Italian-speaking participants were included in the study. They formed an experimental and control group. The experimental cohort consisted of 24 children with ASD aged between 7 and 11;11 years old (mean 9 years and 3 months; standard deviation, SD, 1.70). They had been diagnosed by expert clinicians. Inclusion criteria included the absence of intellectual disability, brain lesions, or auditory difficulties (see Table 1) but the presence of language impairments as certified by a speech therapist and a performance of at least 1.5 standard deviations below expected means on a test of Non-Word Repetition (Marini et al., 2015). Therefore, all participants with ASD had linguistic impairment (ALI).

**TABLE 1:** Means (and standard deviations) showing demographic data of the two groups of participants and their performance on the Raven’s colored matrices and on the Non-word Repetition task.

	ASD (*N* = 24)	TLD (*N* = 50)
Age	9.25 (1.70)	8.65 (1.54)
Education	3.83 (1.90) – range: 1st–6th grade	3.42 (1.53) – range: 1st–6th grade
Raven	23.25 (8.28)	27.82 (4.27)
Non-word repetition*	12.04 (2.12)	14.70 (0.54)

*The asterisk shows when the group-related difference was significant (p < 0.05). ASD, children with Autism Spectrum Disorder; TLD, children with Typical Language Development.*

The control cohort included 50 participants with Typical Language Development (TLD) aged between 7 and 11;11 years old (mean 9 years and 0 months; SD 1.51). They were selected in order to roughly match two controls for every participant with ASD. Inclusion criteria included a normal performance on Raven’s progressive matrices (Raven, 1938), the non-word repetition subtest of the PROMEA (Vicari, 2007), and on the forward and backward digit spans subtests of the Wechsler Scales (Wechsler, 1993). No learning or language difficulties were reported.

The two groups did not differ on age, education or on performance at Raven’s progressive matrices (see Table 1). As expected, an independent-samples *t*-test confirmed that the cohort with ASD scored lower than controls on the Non-Word Repetition subtest of the “Batteria per la Valutazione del Linguaggio in Bambini dai 4 ai 12 anni” (BVL_4-12, Marini et al., 2015) [*t*(46) = -5.873; *p* < 0.001]. All participants came from middle-class families. The study received institutional ethics approval by the ethics committee of the Research Institute IRCCS “E. Medea”. All parents released their informed consent to the participation of their children to the study and the treatment of the data.

### Procedures of Linguistic Assessment

The linguistic assessment was delivered by trained speech-therapists or developmental psychologists in a quiet room at the Research Centers “Ospedale Pediatrico Bambin Gesù” and “E. Medea” (for children with ASD) or their schools (for children with TD). The linguistic assessment focused on lexical, grammatical, and macrolinguistic skills.

#### Assessment of Lexical Skills

The children’s lexical skills were assessed by administering tasks focusing on lexical production and comprehension. Namely, the children received three subtests of the BVL_4-12 assessing naming, lexical comprehension, and discourse production.

In the **naming** task, children are required to name 67 drawings referring to 51 nouns (divided into 15 semantic categories) and 16 action verbs. These words were carefully selected for their frequency of use in Italian (Very high: 17; High: 23; Low: 27). Each correct answer is assigned 1 point. The maximum score is 67.

In the **lexical comprehension** task participants are required to identify which, among four pictures, best represents the meaning of the word produced by the examiner. The pictures represent a target word (i.e., the meaning of the word produced by the examiner, for example, “cat”), a semantic distracter (e.g., a picture portraying the meaning of a word which is semantically related to the target word; in this case “dog”), a phonological distracter (e.g., a picture portraying the meaning of a word which is phonologically related to the target word; “car”), and an unrelated distracter (e.g., “table”). All target words (31 nouns, 10 verbs, and 1 adjective) have been selected according to their frequency in Italian (4 items with very high, 8 with high, and 30 with a low frequency of use). Each correct answer is assigned 1 point for a maximum score of 42.

The **narrative assessment** was performed by analyzing the speech samples obtained by administering the “Nest Story” description task (Paradis, 1987). The recordings of the story descriptions were transcribed and analyzed by two independent coders according to the procedures described in Marini et al. (2011). Namely, the analysis focused on the participants’ speech rates and percentages of semantic errors, paragrammatic errors, omissions of function words, complete sentences, local and global coherence errors, and lexical and thematic informativeness (please see Appendix A for an example of the scoring procedure). The scoring procedure was performed independently by two raters and then compared. The raters were blind with respect to the fact that the transcripts related to stories produced by children with ASD or TD. An inter-rater reliability analysis using the Kappa statistic was performed to determine consistency among raters. Acceptable inter-rater reliability was defined as *k* ≥ 0.80 (Carletta, 1996; Marini and Urgesi, 2012). The interrater reliability scores for the two raters were constantly high. During the analysis, in a few cases the scorers needed to listen again to the audio recordings to face the residual minor issues that could be easily solved.

As for the assessment of their lexical skills, the analyses focused on Speech Rate (words per minute) and the percentage of Semantic Errors. The **Speech rate** was calculated by dividing the number of words produced by the child by the time spent during narrative production (in seconds, using the following formula: (Words/Time_in_seconds)^∗^60. Semantic errors were assessed in terms of both semantic and verbal paraphasias. A semantic paraphasia was scored whenever a target word had been replaced by a semantically related one [e.g., *Fiore* (in English: *Flower*) instead of *Albero* (in English: *Tree*)]. A verbal paraphasia was scored if the target word had been replaced by a semantically unrelated one [e.g., *Cane* (in English: *Dog*) instead of *Albero* (in English: *Tree*)]. The **percentage of Semantic Errors** was calculated by summing semantic and verbal paraphasias and dividing this value by the number of words produced during the narrative description. This score was multiplied by 100.

#### Assessment of Morphological and Grammatical Skills

The assessment of morphological and grammatical skills included tasks focusing on morphological and grammatical production and comprehension skills. Namely, the children received three subtests of the BVL_4-12 assessing sentence completion, syntactic comprehension, and narrative production.

In the **sentence completion** task children are required to produce grammatically sound sentences by processing verbal derivational and inflectional morphology. After hearing a sentence that provides a model [e.g., *Marco apre la porta* (in English: *Marco opens the door*)], the child is presented with the beginning of a second one [the prompt; e.g., *Anche noi …* (in English: *We also*…)] that (s)he is asked to complete assigning the correct morphemes to the verb [the target; e.g., *Anche noi apriamo la porta* (in English: *We also open the door*)]. The test is made of 14 pairs of model sentences and prompts with different levels of grammatical complexity. The first five sentences assess the ability to process inflective morphology with bound morphemes (e.g., *apriamo*). From the sixth item children are asked to cope with more complex sentences with the use of both derivational and inflective morphology [e.g., Model – *Oggi Maria è aiutata dalla mamma a fare i compiti* (in English: *Today, Maria is helped by her mother to do her homework*); Prompt: *Anche ieri Maria* … (in English: *Even yesterday, Maria* …); Expected response: *Anche ieri Maria è stata aiutata dalla mamma a fare i compiti* (in English: *Even yesterday, Maria was helped by her mother to do her homework*)]. Each correct answer is assigned 1 point with a maximum score of 14.

In the test of **syntactic comprehension**, participants are asked to match each of 40 sentences of increasing grammatical complexity with one of four pictures. The pictures represent the meaning of the sentence uttered by the examiner (the target) and three distracters referring to alternative sentences that differ from the target for the presence of inverted thematic roles or other morphosyntactic alterations. For example, after hearing the sentence *Il bambino che è in bicicletta rincorre la bambina che è a piedi* (in English: *The boy who’s on a bike chases the girl who’s on foot*), the child is shown a sheet with four pictures: one depicting its meaning (target) and three distracters representing: 1. *The girl who’s on a bike chases the boy who’s on foot*; 2. *The boy who’s on a bike chases the girl who’s on a bike*; 3. *The girl who’s on a bike is beside the boy who’s on foot*. Each correct answer is assigned 1 point with a maximum score is 40.

The narrative assessment allowed us to obtain a % of Paragrammatic Errors to words, a % of Omission of Function Words to utterances and a % of Complete Sentences. Paragrammatic errors reflect morphological difficulties that include a misuse of bound morphemes {e.g., ^∗^*Questo è una signora* [in English: “^∗^*this* (masculine) *is a woman* (feminine)”]} and/or function words [e.g., *Il signore sale* ^∗^*con l’albero* (in English: “*The man climbs* ^∗^*with the tree*”)]. The **%Paragrammatic Errors** was calculated by dividing the number of Paragrammatic errors by the number of words and multiplying this value by 100. An omission of function words was scored whenever a child omitted fa unction word that was necessarily requested by the sentence [e.g., ^∗^Ramo si spezza (in English: “^∗^Branch breaks”]. The **% Omission of Function Words** was calculated by dividing the number of such omissions by the number of utterances and multiplying this value by 100. As for the **% of Complete Sentences**, a sentence was considered grammatically complete if all of the arguments required by the verb had been inserted correctly and if no omissions or substitutions of free or bound morphemes were detectable. Therefore this percentage was calculated by dividing the number of complete sentences by the number of utterances and then multiplying this value by 100.

#### Assessment of Macrolinguistic Skills

The macrolinguistic skills were assessed in terms of textual organization and informative content. The former aspect was accounted for by calculating a % of Local Coherence Errors and a % of Global Coherence Errors. Local coherence errors were calculated in terms of topic shifts (occurring when an utterance was abruptly interrupted and the following one introduced new information instead of completing the one left incomplete; e.g., / the man is staring at … / and here he is falling /, where the first utterance remained incomplete as the second one introduced a new argument) and missing referents (i.e., instances of words whose referent was not clear or missing as in the following example: /Here they look at a nest / He climbs…/). The **% of Local Coherence Errors** was calculated by summing instances of topic shifts and missing referents, dividing them by the number of utterances in the speech sample and multiplying this value by 100.

Global coherence errors were calculated in terms of utterances that were tangential, conceptually incongruent with the story, repetitions or simple fillers (see Marini et al., 2011 for a detailed description of such errors). The **% of Global Coherence Errors** was calculated by summing instances of tangential, incongruent, repetitive and filler utterances, dividing them by the number of utterances in the speech sample and multiplying this value by 100.

The informative content of the narrative descriptions was assessed in terms of lexical and thematic informativeness. Lexical informativeness was calculated by counting the amount of Lexical Information Units, i.e., those words that were appropriate from a phonological, grammatical and pragmatic point of view. Hence, phonological, morphological and semantic errors, as well as words contained in tangential, repetitive, filler, or semantically incongruent utterances, were excluded from this count. The **% of Lexical Informativeness** was calculated by dividing the number of lexical information units by the number of words produced during the storytelling and multiplying this value by 100.

Finally, the **% of Thematic Informativeness** for each story was measured by dividing the number of thematic units (i.e., those elements of content portrayed in the picture stimulus) produced in each story by the total amount of thematic units available in that story and multiplying this value by 100.

## Results

### Assessment of Lexical Skills

**FIGURE 1:**
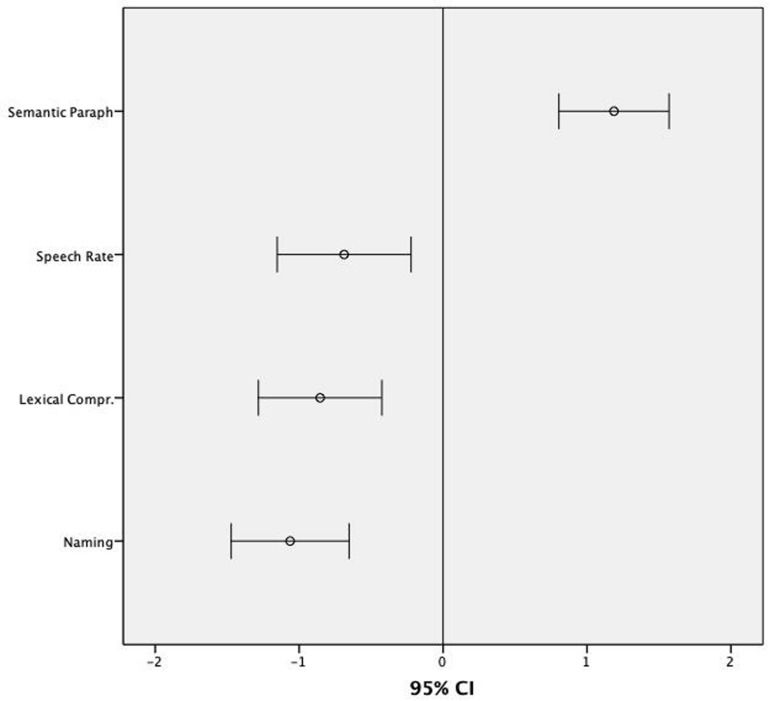
Mean z-scores and 95% CIs for the scores assessing lexical skills in children with ASD in relation to normative data.

The Levene’s test for equality of variances showed that the assumption of homogeneity of variance had been violated for measures of Naming (*p* < 0.001), Lexical Comprehension (*p* < 0.001), Speech Rate (*p* < 0.010), and % Semantic Errors (*p* < 0.001). For this reason, non-parametric Mann–Whitney tests were used to explore between-subject effects on these measures (see Table 2). The level of statistical significance was set at *p* < 0.013 (0.05/4 dependent variables) after Bonferroni correction for multiple comparisons. The group with ASD showed difficulties in all these variables: Naming (*U* = 292.00; *p* < 0.001); Lexical Comprehension (*U* = 371.50; *p* < 0.008); Speech Rate (*U* = 313.50; *p* < 0.001); and % Semantic Errors (*U* = 297.00; *p* < 0.001). Considering a z-score of -1.5 as a cut-off for normality for Speech Rate, Lexical Comprehension and Naming and +1.5 for the production of Semantic Errors, a significant number of participants with ASD scored below normal range in these lexical variables (see Figure 1): 46% in Naming (8% scored -1.5; 38% scored -2); 42% in Lexical Comprehension (4% scored -1.5; 38% scored -2); 25% in Speech Rate (4% scored -1.5; 21% scored -2); 59% in % Semantic Errors (13% scored -1.5; 46% scored -2).

**TABLE 2:** Results of the analysis of lexical skills in the groups of participants with ASD and TLD.

Assessment of lexical skills	ASD	TLD
Naming*	54.21 (8.76)	61.24 (3.81)
Lexical comprehension*	30.71 (8.11)	35.96 (3.46)
Speech rate*	84.10 (41.91)	103.06 (22.24)
% Semantic errors*	2.45 (3.08)	0.39 (0.67)

*Asterisks show when the group-related difference was significant after Bonferroni correction for multiple comparisons (p < 0.013). ASD, children with Autism Spectrum Disorder; TLD, children with Typical Language Development.*

### Assessment of Grammatical Skills

**FIGURE 2:**
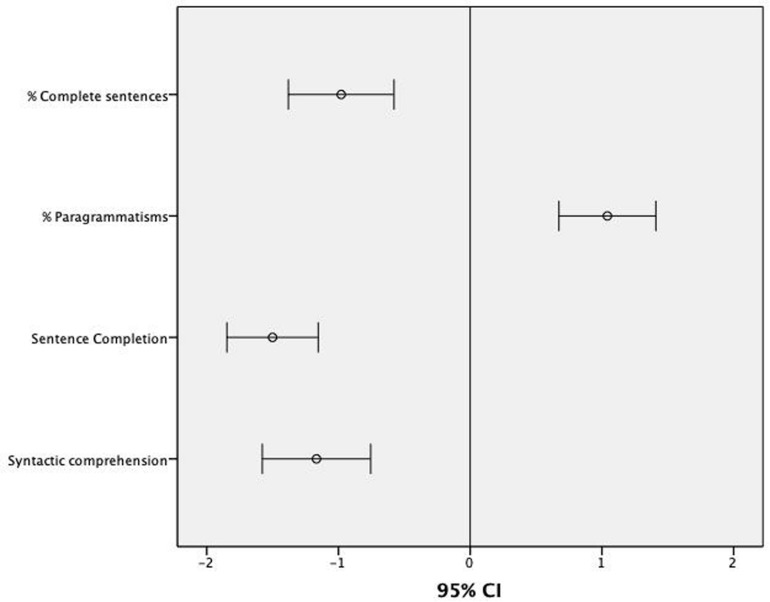
Mean z-scores and 95% CIs for the scores assessing grammatical skills in children with ASD in relation to normative data.

The Levene’s test for equality of variances showed that the assumption of homogeneity of variance had been violated for the measures assessing grammatical skills: Sentence Completion (*p* < 0.001), Syntactic Comprehension (*p* < 0.001), % Paragrammatic Errors (*p* < 0.001), % Omissions of Function Words (*p* < 0.001) and % Complete Sentences (*p* < 0.001). For this reason, a series of non-parametric Mann–Whitney tests with Group (ASD vs. TLD as fixed factor) and the grammatical measures as dependent variables were used to explore between-subject effects (see Table 3). The level of statistical significance was set at *p* < 0.010 (0.05/5 dependent variables) after Bonferroni correction for multiple comparisons. These analyses showed that participants with ASD performed worse than healthy peers in Syntactic Comprehension (*U* = 295.00; *p* < 0.001), Sentence Completion (*U* = 214.00; *p* < 0.001), % Paragrammatic errors (*U* = 293.00; *p* < 0.001), % Omissions of Function Words (*U* = 339.50; *p* < 0.001), and % Complete Sentences (*U* = 312.50; *p* < 0.001). Considering a z-score of -1.5 as a cut-off for normality for Sentence Completion, Syntactic Comprehension, % Paragrammatic Errors and % Complete Sentences and +1.5 for the production of Paragrammatic Errors (normative data for the % of Omissions of Function Words were not available), the majority of participants with ASD scored well below normal range in most of these grammatical variables (see Figure 2): 71% in Sentence Completion (8% scored -1.5; 63% scored -2); 67% in Syntactic Comprehension (25% scored -1.5; 42% scored -2); 51% in % Complete Sentences (13% scored -1.5; 38% scored -2); 54% in % Paragrammatic Errors (25% scored +1.5; 29% scored +2).

**TABLE 3:** Results of the analysis of grammatical skills in the groups of participants with ASD and TLD.

Assessment of grammatical skills	ASD	TLD
Sentence completion*	7.38 (3.92)	11.86 (1.92)
Syntactic comprehension*	30.96 (7.06)	36.18 (2.17)
% Paragrammatic errors*	3.11 (4.04)	0.42 (0.78)
% Omissions of function words*	14.59 (24.58)	0.83 (2.67)
% Complete sentences*	44.34 (29.92)	64.68 (16.70)

*Asterisks show when the group-related difference was significant after Bonferroni correction for multiple comparisons (p < 0.010). ASD, children with Autism Spectrum Disorder; TLD, children with Typical Language Development.*

### Assessment of Macrolinguistic Skills

**FIGURE 3:**
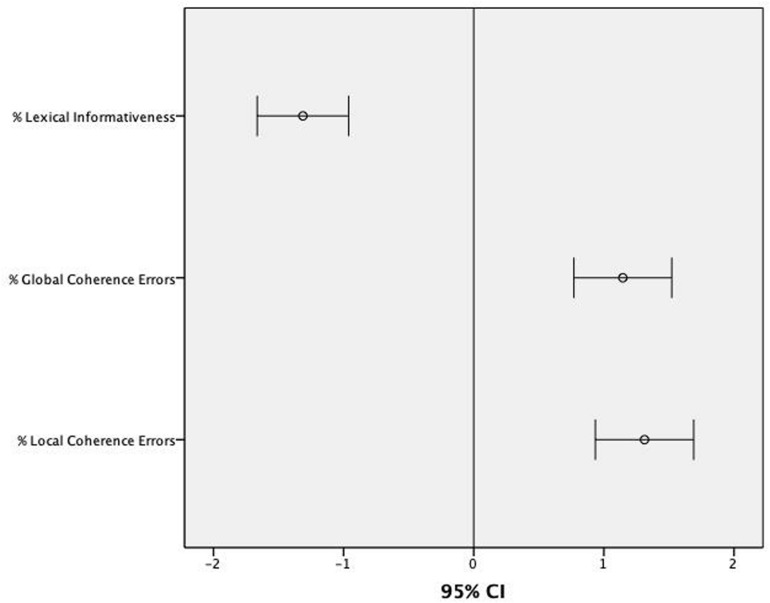
Mean z-scores and 95% CIs for the scores assessing narrative skills in children with ASD in relation to normative data.

As the Levene’s test for equality of variances showed that the assumption of homogeneity of variance had been violated for measures assessing % of Errors of Local (*p* < 0.001) and Global Coherence (*p* < 0.001) and % of Lexical Informativeness (*p* < 0.001) but not for % Thematic Selection (*p* = 0.833), group-related differences on such measures were analyzed with three Mann–Whitney tests for % of Errors of Local and Global Coherence and % of Lexical Informativeness and one independent-samples *t*-test for % Thematic Selection (see Table 4). The level of statistical significance was set at *p* < 0.013 (0.05/4 dependent variables) after Bonferroni correction for multiple comparisons. Table 4 reports the results of these analyses. Overall, the group of participants with ASD produced more errors of Local (*U* = 211.00; *p* < 0.001) and Global Coherence (*U* = 246.50; *p* < 0.001), their narrative samples were characterized by lower levels of lexical informativeness (*U* = 220.00; *p* < 0.001) and Thematic Selection [*t*(72) = -5.493; *p* < 0.001)]. Considering a z-score of -1.5 as a cut-off for normality for % Lexical Informativeness and +1.5 for the production of Local Coherence Errors and Global Coherence Errors (normative data for the % Thematic Selection were not available), the majority of participants with ASD scored well below normal range in most of these macrolinguistic variables (see Figure 3): 63% in % Lexical Informativeness (21% scored -1.5; 42% scored -2); 67% in % Local Coherence Errors (13% scored +1.5; 54% scored +2); 55% in % Global Coherence Errors (13% scored +1.5; 42% scored +2).

**TABLE 4:** Results of the analysis of textual organization and informative content on the narrative production task in the groups of participants with ASD and TLD.

Analysis of textual construction and informative Content	ASD	TLD
% Local coherence errors*	38.81 (31.17)	8.28 (8.61)
% Global coherence errors *	23.49 (15.79)	7.72 (8.77)
% Lexical informativeness*	62.00 (22.19)	83.05 (9.82)
%Thematic selection*	21.18 (12.59)	37.67 (11.84)

*Asterisks show when the group-related difference was significant after Bonferroni correction for muplitple comparisons (p < 0.013). ASD, children with Autism Spectrum Disorder; TLD, children with Typical Language Development.*

### Reassessment of Group Related Differences After Balancing the Two Groups for Number of Participants

As stated in section Participants, the control participants were selected in order to roughly match two controls for every participant with ASD. While providing a quite robust comparison with linguistic skills in children with TLD, this choice might have biased our results because of an unequal number of participants in the two groups. For this reason, the same analyses described in sections Assessment of Lexical Skills, Assessment of Grammatical Skills, and Assessment of Macrolinguistic Skills were re-run after reducing the cohort of control participants by selecting them on the base of their age in order to roughly match one control for every participant with ASD (please see Table 5 for the mean demographic data of the reduced control sample and their performance on the Raven’s colored matrices and on the Non-word Repetition task).

**TABLE 5:** Means (and standard deviations) showing demographic data of the group of controls after the reduction to equal the number of participants in the two groups.

	TLD (*N* = 24)
Age	9.05 (1.51)
Education	3.83 (1.52) – range: 1st–6th grade
Raven	28.54 (4.44)
Non-word repetition*	14.67 (0.12)

*Their performance on the Raven’s colored matrices and on the Non-word Repetition task are also reported. The asterisk shows when the group-related difference was significant (p < 0.05). For scores of the participants with ASD please refer to Table 1. TLD, children with Typical Language Development.*

#### Lexical Skills

Levene’s test for equality of variances showed that the assumption of homogeneity of variance had been violated for measures of Naming (*p* < 0.001), Lexical Comprehension (*p* < 0.001), Speech Rate (*p* < 0.016), and % Semantic Errors (*p* < 0.001). For this reason, non-parametric Mann–Whitney tests were used to explore between-subject effects on these measures (see Table 6). Statistical significance was set at *p* < 0.013 (0.05/4 dependent variables) after Bonferroni correction for multiple comparisons. The group with ASD showed difficulties in all these variables: Naming (*U* = 120.50; *p* < 0.001); Lexical Comprehension (*U* = 138.00; *p* < 0.002); Speech Rate (*U* = 111.00; *p* < 0.001); and % Semantic Errors (*U* = 135.00; *p* < 0.001).

**TABLE 6:** Results of the analysis of lexical, grammatical, and macrolinguistic skills in the reduced group of participants with Typical Language Development.

**Lexical skills**	
Naming*	62.17(2.63)
Lexical comprehension*	37.04(2.85)
Speech rate*	112.01(17.63)
% Semantic errors*	0.29(0.58)

**Grammatical skills**	
Sentence completion*	12.54(1.02)
Syntactic comprehension*	36.96(1.81)
% Paragrammatic errors*	0.44(0.61)
% Omissions of function words*	0.94(2.57)
% Complete sentences *	65.48(12.75)

**Macrolinguistic skills**	
% Local coherence errors*	6.34(6.84)
% Global coherence errors *	6.99(7.63)
% Lexical informativeness*	85.24(6.78)
% Thematic selection*	42.01(13.68)

*Asterisks show when the group-related difference was significant after Bonferroni correction for multiple comparisons. For scores of the participants with ASD please refer to Tables 2–4.*

#### Grammatical Skills

Levene’s test for equality of variances showed that the assumption of homogeneity of variance had been violated for the measures assessing grammatical skills: Sentence Completion (*p* < 0.001), Syntactic Comprehension (*p* < 0.001), % Paragrammatic Errors (*p* < 0.001), % Omissions of Function Words (*p* < 0.001), and % Complete Sentences (*p* < 0.001). For this reason, non-parametric Mann–Whitney tests with Group (ASD vs. TLD as independent variable) and the grammatical measures as dependent variables were used to explore between-subject effects (see Table 6). Statistical significance was set at *p* < 0.010 (0.05/5 dependent variables) after Bonferroni correction for multiple comparisons. These analyses revealed that individuals with ASD performed worse than healthy peers in Syntactic Comprehension (*U* = 112.50; *p* < 0.001), Sentence Completion (*U* = 78.50; *p* < 0.001), % Paragrammatic errors (*U* = 145.00; *p* < 0.002), % Omissions of Function Words (*U* = 166.50; *p* < 0.002), and % Complete Sentences (*U* = 147.00; *p* < 0.004).

#### Macrolinguistic Skills

As Levene’s test for equality of variances showed that the assumption of homogeneity of variance had been violated for measures assessing % of Errors of Local (*p* < 0.001) and Global Coherence (*p* < 0.001) and % of Lexical Informativeness (*p* < 0.001) but not for % Thematic Selection (*p* = 0.532), group-related differences on such measures were analyzed with three Mann–Whitney tests for % of Errors of Local and Global Coherence and % of Lexical Informativeness and one independent-samples *t*-test for % Thematic Selection (see Table 6). Statistical significance was set at *p* < 0.013 (0.05/4 dependent variables) after Bonferroni correction for multiple comparisons. As reported in Table 4, the group of participants with ASD produced more errors of Local (*U* = 87.50; *p* < 0.001) and Global Coherence (*U* = 110.50; *p* < 0.001), their narrative samples were characterized by lower levels of lexical informativeness (*U* = 85.00; *p* < 0.001) and Thematic Selection [*t*(46) = -5.491; *p* < 0.001].

### Do Morphological Difficulties Relate to Grammatical Impairments in Children With ASD and Controls?

A goal of this study was to determine whether the morphological difficulties often observed in children with ASD are related to grammatical (i.e., morphosyntactic and syntactic) difficulties while producing samples of narrative language. This was explored by performing a series of correlational analyses between the children’s performance on the test assessing Sentence Completion and the other grammatical variables obtained with traditional tasks (i.e., Syntactic Comprehension) and narrative analysis (i.e., % Omission Function Words and % Complete Sentences). These analyses showed that, in children with ASD, the performance on the sentence completion task correlated with all of the above-mentioned variables: Syntactic Comprehension (Spearman’s Rho = 0.797; *p* < 0.001), % Complete Sentences (Spearman’s Rho = 0.517; *p* < 0.010), % Omission Function Words (Spearman’s Rho = -0.483; *p* < 0.017).

On the contrary, the performance of participants with TLD on the sentence completion task did not correlate with any of the above-mentioned variables: Syntactic Comprehension (Spearman’s Rho = 0.002; *p* = 0.992), % Complete Sentences (Spearman’s Rho = -0.395; *p* = 0.056), % Omission Function Words (Spearman’s Rho = -0.057; *p* = 0.793).

### Are Macrolinguistic Disturbances Related to Microlinguistic Difficulties in Children With ASD and Controls?

The possibility that macrolinguistic disturbances (i.e., % of Local and Global Coherence Errors) might be related to the microlinguistic difficulties (i.e., measures of lexical and grammatical skills) was explored by using Spearman’s Rho correlation coefficient. In the group of children with ASD both % Global and % Local Coherence Errors were negatively correlated to the % Complete Sentences (Global Coherence Errors: Spearman’s Rho = -0.477; *p* < 0.018; Local Coherence Errors: Spearman’s Rho = -0.430; *p* < 0.036).

On the contrary, in participants with TLD the % Complete Sentences did not correlate with the production of Global (Spearman’s Rho = -0.193; *p* = 0.366) or Local Coherence Errors (Spearman’s Rho = -0.070; *p* = 0.745).

## Discussion

This study investigated linguistic and narrative abilities in a cohort of children with ASD and language impairments. The linguistic assessment was performed with both traditional tests and a multilevel procedure for discourse analysis. Overall, analyses involving both the complete sample of participants (*N* = 74 with 50 controls) and the reduced number of participants (*N* = 48 with 24 controls) showed that the children with ASD had significant lexical, grammatical and narrative difficulties. A series of correlational analyses confirmed that (1) morphological difficulties were related to the observed grammatical impairments; (2) global coherence errors were negatively correlated to the production of complete sentences.

Not surprisingly, the participants with ASD and language impairments showed significant narrative difficulties. Indeed, 67% and 65% of them produced a significant amount of local and global coherence errors (see also Kuijper et al., 2017) that likely contributed to the reduction of their levels of lexical informativeness. Indeed, their narratives were characterized by the inclusion of repetitive and overtly incoherent utterances that were quite often not correctly linked with each other (e.g., Diehl et al., 2006; Baixauli et al., 2016; Volden et al., 2017). Of note, they also produced fewer ideas that were portrayed in the vignettes as reflected by the reduced % of Thematic Selection. This last finding may suggest that a significant difficulty was in the phase of non-verbal conceptualization of the story. According to the Structure Building Framework (Gernsbacher, 1990; see Marini et al. (2017) for its application in the domain of narrative production) the generation of a narrative discourse relies on a multistage process. In the first stage, prelinguistic, it is necessary to generate a mental model or scenario of the story that will serve as a foundation for its development. As the information flows, the speaker needs to continuously monitor the consistency of such mental models and scenarios with the generated structures. In case of inconsistency, it becomes necessary to generate new structures that are in line with the desired mental model. These will eventually trigger the generation of propositions organized at the macrolinguistic level through adequate coherent and cohesive links among the utterances. The macrolinguistic impairments of participants with ASD were likely related to more general difficulties in the prelinguistic conceptual phase of message planning. Namely, they might stem from cognitive difficulties affecting those executive functions that are required to adequately plan a discourse structure, monitor its production, and inhibit the potential production of utterances that are not coherent with the flow of the story (see also Miyake et al., 2000; Mozeiko et al., 2011). Interestingly, in the group of children with ASD both global and local coherence errors negatively correlated with the % of complete sentences suggesting the possibility that an inability to generate a correct mental model of the story induces the production of utterances that are not coherent with the story that, in turn, may frequently trigger a pause in the subsequent phase of grammatical construction. These correlations suggest that the participants’ grammatical difficulties were related to their macrolinguistic impairments and support the hypothesis that their difficulties in message planning and organization might have an impact on their grammatical production skills.

According to an influential model (e.g., Levelt, 1989; Levelt et al., 1999), message production is a complex activity that requires different processing stages. The first is a phase of message planning. Here, speakers need to generate the conceptual organization of the message, which includes also the formulation of an internal representation of the addressee’s mental model and the use of such representation to subsequently select words with unambiguous referents. This is an area of particular weakness for individuals with ASD. Indeed, even when they can take to some extent the interlocutor-specific prior experience into account, they may produce words whose referent is not always clear to their listeners (e.g., Arnold et al., 2009; Banney et al., 2015; Malkin et al., 2018). Our data confirm this weakness by showing the presence of a significant amount of a specific type of error of local coherence, i.e., the production of words with ambiguous referents. Therefore, the communicative inefficacy of their narrative samples stems, at least in part, from such inability to use nouns and or pronouns to unambiguously refer to elements of the story. After the phase of message planning, it is necessary to extract lexical concepts from memory and this eventually triggers a phase of lexical selection. At this stage, the identification of the right word can be obtained thanks to the inhibition of potential semantic competitors. As to this regard, the participants with ASD had also lexical retrieval difficulties. Indeed, half of them had clinically significant difficulties in naming (46%) and lexical comprehension (42%). Furthermore, such difficulties were reflected also in a reduced narrative fluency (25% of the participants with ASD had reduced speech rates) and the production of semantic errors (in 59% of the cases). These results suggest that the process of lexical selection is impaired in both modalities (i.e., production and comprehension) in this group of individuals with ASD. Therefore, a difficulty in the process of lexical selection might explain their performance on measures tapping also lexical skills. However, as already observed for their macrolinguistic difficulties, this does not necessarily imply that such difficulties arise from a purely linguistic impairment. Indeed, the ability to select the target lexical item in the mental lexicon requires also additional cognitive skills, such as working memory, attention, and executive functions (e.g., inhibition, monitoring, and planning; Miyake et al., 2000; Mozeiko et al., 2011). For example, the production of semantic paraphasias may stem from a failure in the activation of the right lexical item because of the interferences provided by the semantic competitors. Unfortunately, in this study we did not control for such non-linguistic variables. This is a limitation that should be addressed by future studies.

Notably, according to the model of message production by Levelt et al. after selecting the target word the speaker has access to the information stored in it. During the phase of access to the word’s lemma, (s)he becomes aware of the morphosyntactic structure required by the word and will use this information to put the selected item in the right position in the sentence. Furthermore, in the phase of morphological coding (s)he will get access to the morphological information regarding the word. The ASD participants’ performance on the sentence completion subtest of the BVL_4-12 and the enhanced production of morphological errors (i.e., % of paragrammatic errors) and omissions of function words in their narrative speech samples suggest that they had also difficulties in the phases of access to morphosyntactic and morphological information. Indeed, the majority of these participants had clinically significant difficulties in such measures: 71% in sentence completion; 67% in syntactic comprehension; 59% in the production of paragrammatic errors; and 51% in the production of complete sentences. These results agree with previous investigations. They highlight the production of utterances characterized by instances of pronoun reversal in ASD (e.g., Kuijper et al., 2017) as well as the presence of persisting morphological difficulties in school-age children with ALI ranging from 6 years up (Roberts et al., 2004; see also Kuijper et al., 2017). Notably, our results suggest that the participants’ morphological and morphosyntactic difficulties likely affected their grammatical skills as also shown in previous investigations (e.g., Tuchman et al., 1991; Eigsti et al., 2007). This relation between morphological, morphosyntactic and grammatical difficulties is supported not only by the reduced number of complete sentences on the narrative production task and their impaired performance on the sentence completion and syntactic comprehension tasks. The correlational analyses support such relation. Indeed, their morphological difficulties were related to the production of fewer grammatically well-formed sentences in the narrative production task (see also Condouris et al., 2003; Losh and Capps, 2003).

In conclusion, the results from the current study support the claims about the generalized linguistic difficulties in these children (e.g., Boucher, 2012). As can be seen in Appendix B, even if these difficulties are associated to a large within-group variability (e.g., Rapin and Dunn, 2003), 79% of the participants (19 out of 24) performed below normal range in several measures tapping lexical, grammatical and narrative skills, highlighting difficulties at different stages of message planning, organization, and microlinguistic (i.e., lexical and grammatical) processing. Their macrolinguistic impairments were likely related to more general difficulties in the prelinguistic conceptual phase of message planning and mental model generation of the story. Such weaknesses included a difficulty in the non-verbal conceptualization of the story and the generation of an internal representation of the addressee’s mental model triggering the production of stories with violations of both local (i.e., production of words with ambiguous referents) and global coherence that significantly contributed to the reduction of the levels of lexical informativeness. Furthermore, the majority of participants with ASD showed also difficulties on tasks assessing lexical selection and grammatical processing skills in both modalities (i.e., production and comprehension). Apparently, only five individuals (21%) showed spared lexical and morphosyntactic skills. However, two of them diverged significantly from the norms in the production of semantic paraphasias, one produced too many paragrammatic errors, and only two did not show any difficulty at the narrative level. Furthermore, only 2 of the 19 participants with ASD with linguistic difficulties did not show any difficulty in comprehension, whereas the rest of the cohort shoed mixed receptive-expressive disorders. Overall, these findings are in line with previous investigations showing similarities in the language impairments observed children with Developmental Language Disorders (e.g., Williams et al., 2008; Bishop et al., 2017) and have both clinical and research implications. From a clinical point of view, they support the efficacy of non-word repetition tasks in detecting the presence of linguistic difficulties in children with ASD (e.g., Kjelgaard and Tager-Flusberg, 2001). They also support the need to adequately assess the linguistic profile of children with ASD by administering not only traditional tasks but also narrative production tasks that allow clinicians to have a clearer picture of the real linguistic skills of persons with ASD and the interconnections between different stages of message production (e.g., phases of message planning, lexical selection, lexical access, etc…). Furthermore, a comprehensive assessment should include also other cognitive skills that may affect narrative processing. For example, in line with previous studies focusing on the potential role of executive functions in narrative discourse (e.g., Miyake et al., 2000; Mozeiko et al., 2011), we speculated that the difficulties in the prelinguistic phase of message planning and conceptual organization might stem from executive functions’ difficulties (i.e., impairments in the ability to plan a discourse structure, monitor its production, and inhibit the potential generation of utterances that are not coherent with the flow of the story). However, such measures were not available in the current investigation. Future studies should include also measures assessing executive functions and recruit larger numbers of participants to run regressions models that might allow both researchers and clinicians to explore the proposed causative relation between executive difficulties, macrolinguistic disorganization and microlinguistic impairment in ASD.

## Data Availability Statement

The datasets presented in this article are not readily available due to confidentiality reasons. Requests to access the datasets should be directed to the corresponding author.

## Ethics Statement

The study received institutional ethics approval by the Ethics’ Committee of the Research Institute IRCCS “E. Medea.” Written informed consent to participate in this study was provided by the participants’ legal guardian/next of kin.

## Author Contributions

AM planned the study, ran the statistical analyses, and wrote the manuscript. GV supervised the recruitment of the participants. RM administered the tasks to the children. MO contributed to the analyses. All authors contributed with comments to the interpretation of the results.

## Conflict of Interest

The authors declare that the research was conducted in the absence of any commercial or financial relationships that could be construed as a potential conflict of interest.

## Figures and Tables

**TABLE A1:**
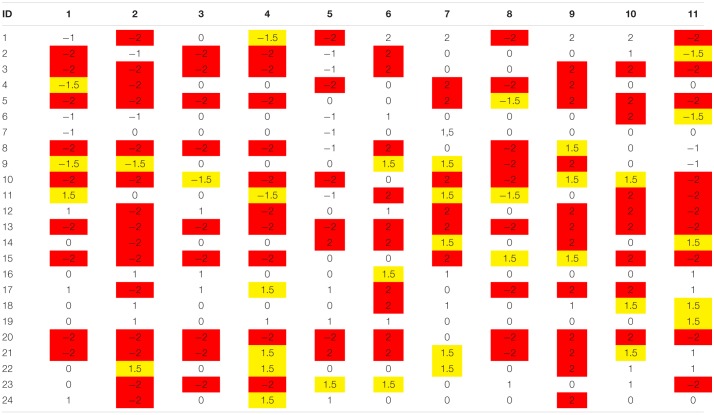
Table detailing the performance of each child with ASD on each linguistic measure

*Table showing the z-scores of each participant with ASD on each linguistic measure. Z-scores highlighted in yellow show performances of about 1.5 SDs above (in case of errors) or below expected means. Z-scores highlighted in red show performances of about at least 2 SDs above (in case of errors) or below expected means. Legend: 1. Naming; 2. Sentence Completion; 3. Lexical Comprehension; 4. Grammatical Comprehension; 5. Speech Rate; 6. % Semantic Errors; 7. % Paragrammatic Errors; 8. % Complete Sentences; 9. % Local Coherence Errors; 10. % Global Coherence Errors; 11. % Lexical Informativeness.*

## Appendix A

### An Example of the Narrative Analysis Performed on a Description of the Nest Story

Subject C.S. / Group: ASD / Age: 9 years; 7 months / Gender: Male.

**Italian version (original) with errors marked in English:**

“Loro^*missing referent*^ **vedono il nido di uccelli** / **poi** *…(3 seconds)* / due persone^*repetition of* 2 *words + repetition of utterance*^ / due persone^*repetition of* 2 *words + repetition of utterance*^ / due persone vedono il nido^*repetitionof*5*words* + *repetitionofutterance*^ / una^missing referent^ **diceva** / “**puoi prendere il nido** sotto^*paragrammatic error*^ ” / **allora la** persona^missing referent^ **prendeva il nido** / Luca^missing referent^ scappa^*semantic paraphasia*^ / poi^*filler*^ **a un certo punto** alcune^missing referent^ vedeva^*paragrammatic error*^ / **dicevano** / si^*missing referent*^ **era fatto malea+lla gamba** / **poi una** persona^*missing referent*^ **era triste** / ^*missing referent*^ **è andata in ospedale**…/ una persona era triste^*repetition of* 4 *words + repetition of utterance*^ / lui^*missing referent*^ **era quasi svenuto /**”.

Time 40”.

NB In bold the informative words. The complete sentences are underlined.

**English translation:**

“They see the nest with the birds / then …(3 seconds) / two people / two people / two people see the nest with the birds / one said / “can you take the nest under” / then the person took the nest / Luca runs away *[falls]* / then at some point some seen / said / he had hurt his leg / then a person was sad / went to the hospital / a person was sad / he was almost unconscious /”.

**Narrative analysis:**

Words: 63 / Utterances: 16 / Speech Rate: 95 words per minute / % Semantic Errors: 1.6 % / % Paragrammatic Errors: 3% / % Complete Sentences: 63% / % Local Coherence Errors: 56% / % Global Coherence Errors: 25% / % Lexical Informativeness: 60%.
